# How to derive ethically appropriate recommendations for action? A methodology for applied ethics

**DOI:** 10.1007/s11019-022-10133-9

**Published:** 2022-12-15

**Authors:** Sebastian Schleidgen, Alexander Kremling, Marcel Mertz, Katja Kuehlmeyer, Julia Inthorn, Joschka Haltaufderheide

**Affiliations:** 1 Institute of Philosophy, FernUniversität in Hagen, Hagen, Germany; 2 Institute for History and Ethics of Medicine, Martin Luther University Halle-Wittenberg, Halle, Germany; 3 Institute of Ethics, History and Philosophy of Medicine, Hanover Medical School, Hanover, Germany; 4 Institute of Ethics, History and Theory of Medicine, Ludwig-Maximilians-University Munich, Munich, Germany; 5 Center for Health Care Ethics, Hanover, Germany; 6 Faculty of Health Sciences, University of Potsdam, Potsdam, Germany

**Keywords:** Bridge principles, Contingency, Empirically informed ethics, Morally relevant situations

## Abstract

Researchers in applied ethics, and some areas of bioethics particularly, aim to develop concrete and appropriate recommendations for action in morally relevant real-world situations. When proceeding from more abstract levels of ethical reasoning to such concrete recommendations, however, even with regard to the very same normative principle or norm, it seems possible to develop divergent or even contradictory recommendations for action regarding a certain situation. This may give the impression that such recommendations would be arbitrary and, hence, not well justified. Against this background, we, first, aim at showing that ethical recommendations for action, although being contingent in some sense, are not arbitrary if developed appropriately. For this purpose, we examine two types of contingencies arising in applied ethics reasoning based on recent examples of recommendations for action in the context of the COVID-19 pandemic. In doing so, we refer to a three-step model of ethical reasoning towards recommendations for actions. This, however, leaves open the question of how applied ethics may cope with contingent recommendations for action. Therefore, in a second step, we analyze the role of bridge principles for developing ethically appropriate recommendations for action, i.e., principles which connect normative claims with relevant empirical information to justify certain recommendations for action in a given morally relevant situation. Finally, we discuss some implications for reasoning and reporting in empirically informed ethics.

## Introduction

Researchers in applied ethics aim at developing recommendations for action based on ethical argumentation. Such recommendations are suggestions of what ought to be done in a specific morally relevant situation. Consider the following three recommendations for action in the context of the COVID-19 pandemic:

1. In the current COVID-19 pandemic, nurses should engage in epidemiological screening and frailty assessment to identify high-risk groups and minimize transmission (Andrew et al. 2020; Dosa et al. 2020; Gnanasambantham et al. 2021).
2. In the current COVID-19 pandemic, nurses should use technological devices for telehealth to reduce their direct contact with residents (Cormi et al. 2020).
3. In the current COVID-19 pandemic, nurses should engage in communication and care planning with patients and relatives with regard to the current situation (Gaur et al. 2020).

Recommendations like (1)–(3) can be understood to have some normative point of reference, to be guided by some general moral principle, norm or maxim (Musschenga 2005; Düwell 2009; Salloch et al. 2012). To develop recommendations for action, the normative content of such general principles, norms, or maxims must, first, be specified and, second, be contextualized with a view to relevant empirical knowledge about the specific situation at hand. Developing ethically appropriate recommendations for action, thus, consists of three major conceptual steps: first, the explication of a general principle (providing a normative point of reference); second, the specification of its normative content with the aim of deriving what we call *moral judgments;* and third, the empirically informed contextualization of such judgments in view of the specific morally relevant situation and context at hand. Proceeding from the first to the second level, hence, is to clarify what ought to be done under certain conditions given a certain basic moral principle or general norm, while proceeding from the second to the third level means translating this prescription into practice and to adapt it to a certain situation or context.

This may lead to the impression that we implicitly argue for a deductivist and foundationalist form of moral reasoning. This would, however, be incorrect. On the contrary, our claim also holds for concepts of ethical reasoning that are explicitly non-deductivist or at least non-foundationalist. Casuistry, for example, arrives inductively at more abstract rules or maxims, based on paradigmatic cases and analogical reasoning. Nonetheless, moral obligations regarding specific situations are “framed in terms of rules or maxims that are general but not universal or invariable” (Jonsen and Toulmin 1988). Accordingly, even in casuistic approaches, general rules or maxims must be explicated, specified and contextualized to arrive at moral recommendations like (1)–(3). Although explication proceeds via case comparison and abstraction, the conceptual step of establishing general rules or maxims is still part of such approaches. Furthermore, when applying more abstract rules or maxims to concrete individual cases, the steps of specification and contextualization remain necessary—even if these steps may be tied back to an identification of relevant analogies and disanalogies to the underlying paradigmatic cases. The three steps of explicating, specifying and contextualizing general principles, norms or maxims are also part of non-foundationalist strategies of justification such as coherentist approaches. It is true that general principles, moral judgments as well as their empirical contextualization, according to such accounts, are each epistemically non-privileged beliefs in a belief system aiming at sufficient coherence. Sufficient coherence, however, alludes to the claim that abstract principles, moral judgments and their empirical contextualization mutually justify each other (sufficiently), which, in turn, presupposes our three conceptual steps (possibly in the sense of mutual adjustment steps).

As these examples show, we do not deny that there are considerable differences in how the three steps are carried out as this depends on underlying metaethical presuppositions. However, we are only concerned with presenting a *model*. As with every model its sole purpose is to represent *certain aspects* of reality. We do not claim that this is the only way to represent the process of ethical reasoning but just that it is a fruitful one. In addition, we do not claim that our model depicts the complete process of developing ethically appropriate recommendations for action. We rather think of it as representing *one* aspect. We do think, however, that this aspect is important and might be part of nearly every form of ethical reasoning. We, hence, just want to advance the claim that any ethical theory or approach that aims to develop recommendations for action, i.e. suggests what ought to be done in a specific situation, has to process the three steps outlined above.

However, one might argue that the crucial connection between the different levels of developing ethically appropriate recommendations for action often seems to be underdetermined or not even considered. For instance, it is at least unclear as to what basic moral principle, general norm, or moral judgments the recommendations (1)–(3) refer. On the other hand, however, it is often possible to derive differing recommendations for action from the very same basic moral principle or moral judgment. In fact, (1)–(3) could (implicitly) refer to the very same basic moral principle or moral judgment as will be shown later. Hence, developing ethically appropriate recommendations for action seems to face the problem that links between proposed recommendations for action and the underlying moral judgments remain unclear (as do links between moral judgments and basic moral principles); rather, these links seem to be, at least to some degree, *contingent*: there is usually no sole recommendation for action that *necessarily* follows from a proposed moral judgment and no sole moral judgment that *necessarily* follows from a proposed basic principle.

On the surface, such contingency may not only lead to divergent, but eventually even to contradictory recommendations being established in view of the very same morally relevant situation and against the background of the very same moral judgment(s) and/or basic moral principle(s) or norm(s). For instance, if (1)–(3) in fact referred to the very same basic moral principle as well as the very same moral judgment, they could be contradictory in the sense that implementing at least one of the recommendations would necessarily exclude implementing at least one of the remaining recommendations.

This has some serious implications. From a theoretical perspective, ethical reasoning aims at developing arguments by principles of reason and thought. This raises the question of the significance of contingencies regarding the possibility of assessing ethical arguments according to their soundness, validity, or strength.

Furthermore, as applied ethics is concerned with developing recommendations that are applicable in practice, contingencies and their unclear status may also affect and undermine translation into practice by suggesting a certain arbitrariness of ethical recommendations for action. Particularly, when it comes to the implementation of ethical guidelines, the inability to explain and cope with contingent recommendations *eo ipso* presents a barrier to come to a well-justified agreement. Consider the ongoing COVID-19 pandemic, which raises a wide range of serious issues asking for ethically appropriate recommendations for action (e.g., handling triage situations, distributing vaccines, or moral obligations of caregivers in situations of personal risk). Such recommendations must consider the relevant empirical knowledge regarding, e.g., the properties of the virus, prospective long-term consequences of recommended actions or their psychological and social effects. Besides the empirical state of knowledge changing almost daily, divergent, eventually contradictory recommendations for action (like, e.g., (1)–(3)) might give the impression of a normatively undecided situation. This shows the paramount importance of clarifying the links between the different levels of developing ethically appropriate recommendations for action (recommendations for action, moral judgments, and basic moral principles) as well as the empirical information referred to: for this would allow understanding, evaluating, and critiquing the specific ways in which the respective empirical reference points are used to infer certain recommendations for action within an ongoing debate.

Upon closer examination, we find the reason for the contingencies introduced so far in additional non-trivial premises which are needed to arrive at (and/or justify) moral judgments via basic moral principles as well as to arrive at (and/or justify) ethically appropriate recommendations for action via these moral judgments. The latter is of particularinterest for applied ethics. It refers to premises specifying how to bridge *what ought to be done*—exemplified by a moral judgment—and *what is* (i.e., the respective (set of) empirical premises).^1^ Reference to differing premises (which, moreover, mostly remain implicit), however, leads to the contingency of ethically appropriate recommendations for action derived from the very same set of moral judgments as well as empirical premises. If this holds true, such contingent recommendations are not necessarily arbitrary and can indeed be well-justified.

In what follows, we aim at underpinning this claim by shedding light on the problem of contingency arising at the different stages of developing ethically appropriate recommendations for action. For this purpose, first, we outline a three-step model of ethical reasoning towards recommendations for action. Second, we address two types of contingencies arising in the context of this model. Third, we highlight the role of bridge principles as non-trivial additional premises that connect moral judgments and empirical knowledge. Finally, we discuss some important implications of our considerations for ethical research and reporting.

## A model of ethical reasoning in applied ethics

We start from the assumption that one of the main tasks of applied ethics can be reconstructed as the application of basic moral norms and principles to solve concrete morally relevant issues in specific contexts. This means to develop concrete recommendations for action, i.e., to develop statements denoting specific realizable actions within certain contexts that prescribe what is (from an ethical perspective) deemed to be the right thing to do. For this purpose, three main conceptual steps are necessary. First, a basic moral principle or a general norm needs to be explicated. We refer to this as the A-level of ethical reasoning.^2^

A crucial problem is that basic moral norms and principles are, loosely speaking, too abstract to allow for establishing or identifying concrete recommendations for action in view of a specific situation. This is mainly due to their generality and absoluteness. *Generality*, loosely referring to Gewirth (1981), concerns the range of applicability of a prescriptive statement, i.e., to the question of which moral subjects should be guided by a moral norm or principle. *Absoluteness*, on the other hand, refers to the degree of a moral obligation. For instance, only very few obligations apply to any moral agent (regarding generality) and without any exception (regarding absoluteness). Some apply as permission towards some agents, some as prohibitions and some may be trumped by other obligations. Nevertheless, basic moral principles or general norms usually imply moral obligations of high generality as well as a high degree of absoluteness.

Against this background, it becomes clear that further steps are necessary to arrive at recommendations for action. In a second step of ethical reasoning, the generality and absoluteness of a basic moral norm or principle must be reduced to *specify* its prescriptions with a view to the context at hand. This results in *moral judgments* on the B-level of ethical reasoning, which *conceptually* specify the moral obligations formulated by a moral norm or principle on the A-level in view of certain general conditions, i.e., the general circumstances in which a norm or principle is to be applied.^3^ Such circumstances may be, for instance, conditions of poverty, environmental degradation, resource scarcity in health care systems, or pandemic situations. With a view to this, a basic moral principle needs to be specified in at least five dimensions in order to develop moral judgments: against the background of the initial normative standard, it has to name an addressee and a subject of moral significance or bearer of rights. It, then, has to specify the nature of the prescription, that is, the degree of obligation, permission or prohibition. Finally, it needs to detail the conditions under which an action is prescribed. In short, this includes to answer the questions of (1) who has to consider (2) whose moral rights to (3) what degree of obligation, permission, or prohibition (4) under certain conditions against the background of (5) a certain normative standard (exemplified by the basic moral norm or principle on the A-level).

However, defining a specific and realizable action is beyond the scope of moral judgments. To arrive at specific recommendations for action, it is, in a third step, required to include relevant information on the contextual determinants of a morally relevant situation. We understand a recommendation for action to be a normative statement that *contextualizes* the content of a moral judgment in view of a specific temporal and spatial context and, in addition, recommends a specific action that is realizable within this context (Dietrich 2009). Hence, recommendations in addition to providing rather general answers regarding the five dimensions of moral judgments aim at substantiating these aspects with a view to concrete real-world situations and contexts under the general conditions which motivated developing the underlying moral judgments. Recommendations for action thus aim at dealing with specific real-world problems in specific contexts and at suggesting courses of action that correspond to the obligations expressed in moral judgments. They are developed with a view to at least six questions: (1) who has to consider (2) whose moral rights (3) *in which way* to (4) what degree of obligation, permission, or prohibition (5) *in a specific context* against the background of (6) a certain normative standard (exemplified by a moral judgment on the B-level). Hence, when speaking of recommendations for action—in contrast to moral judgments—the additional dimension (3) is implied. Furthermore, as dimension (5) makes clear, it is not certain general conditions that are relevant for developing recommendations for action (as specified in dimension (4) of moral judgments), but rather specific contexts in which they are applied. Regarding the question of realizability, it is furthermore important to know, for instance, who actually can exercise what kind of action, what different measures might be available or what their effects and outcomes are. This, ultimately, is a matter of social organization, professional roles and responsibilities, causal effects, availability of resources and other, even more complicated issues. This third step results in what we call the C-level of ethical reasoning and requires extensive empirical knowledge on the actual context and needs to review what ought to be done in the light of what is.

## Moral principles, moral judgments, and recommendations for action: two contingencies

This model exposes a problem that arises in the transition between different levels of ethical reasoning. Consider the different attempts to deal with the risks arising in the COVID-19 pandemic for residents of nursing homes. Furthermore, consider the basic moral principle that a person’s wellbeing is worthy of appropriate consideration:

- Every person’s wellbeing is to be promoted.

(a) represents the A-level of ethical reasoning. For the purpose of our argument, we will not define “wellbeing” further than necessary. For instance, we will leave open the question of whether wellbeing is best explained by hedonist, desire-satisfaction, or objective list theories (Parfit 1987). We assume, however, that “wellbeing” refers to the conditions for developing the affective, cognitive, and emotional capacities necessary for living a good life. We further assume that these—at least to a certain extent—include physical and mental health.

Obviously, many different moral judgments may count as a specification of a basic moral principle like (a) with a view to certain general conditions. One could, for instance, reasonably argue that (a) can be specified in ways that result in the following moral judgments:

1. In a global pandemic situation, nurses should promote residents’ wellbeing.
2. In a global pandemic situation, relatives are obliged to promote the wellbeing of family members living in nursing homes.
3. In a global pandemic situation, nurses are obliged to promote residents’ freedom of physical pain.
4. In a global pandemic situation, nurses are obliged to promote residents’ mental health.
5. In a global pandemic situation, relatives should promote freedom of pain of family members living in nursing homes.
6. In a global pandemic situation, relatives should promote mental health of family members living in nursing homes.

(b1-6) represent the B-level of ethical reasoning, i.e., they specify (a) in the context of a global (e.g., COVID-19) pandemic and with a view to the situation of residents of nursing homes. According to Paulo (2012), the relation of moral principles and specified instances of these principles is a “purely formal” one. The notion of “purely formal” denotes that it is to be understood as a relation between a class and its instances. While moral norms and principles, by defining general obligations to be realized as far as possible, constitute the defining properties of such classes, moral judgments constitute instances of these general obligations that satisfy their requirements given certain general conditions. However, as the examples of (b1-6) show, the process of specification may give rise to different moral judgments, with also differing degrees of abstractness or concreteness. However, not only are complementing judgments conceivable (as (b1-6) could be), but also contradictory ones. For instance, the claim that nurses are obliged to promote residents’ freedom of physical pain (b3) could, under certain circumstances, contradict the claim that nurses are obliged to promote residents’ mental health (b4), for the former could imply that nursing staff should reduce direct contact with residents to minimize the risk of infection whereas the latter may imply that contact should be increased to avoid emotional distress (however, this is not clear at the level of moral judgments).

However, it is not possible to rank such judgments according, e.g., to their moral value. Rather, any of the judgments (b1-6) must be *prima facie* considered equally valid with a view to the underlying basic moral principle (a) (if the relation between (a) on the one hand and the judgments (b1-6) on the other is formally correct). This is what we call *contingency*_*1*_, designating the relationship between a moral principle and related moral judgments as not being necessary but not being impossible (and therefore not giving decisive reason to comply with a certain judgment). In other words, the relation between a moral principle and a certain moral judgment is contingent insofar as a moral judgment satisfies the general obligations of a basic moral principle (i.e., it is an instance of a certain class) but is not the only moral judgment conceivable that satisfies these very obligations (i.e., the given class has more than one instance). Thus, additional reasons must be given with a view to the question of why a certain judgment is preferable over others.

On some occasions of contingency_1_, it might be possible to give purely theoretical reasons for one judgment being preferable, that is reasons for treating certain judgments as not being an instance of a given class. On other occasions, it might be the case that some moral judgments can be ruled out by competing moral principles, for example, by showing that they would violate other basic moral obligations.

(b1-6), however, are not recommendations for action. As regards (b3), for instance, it is not yet clear whether it implies that nursing staff should reduce direct contact with residents to minimize the risk of infection. As regards (b4), it remains open whether it implies that contact should be increased to avoid emotional distress. To arrive at recommendations for action it is necessary to further concretize moral judgments like (b1-6). For instance, it is possible to comply with (b3) in the three different ways mentioned in the introduction above by reference to differing contextualization, e.g.:^4^

1. In the current COVID-19 pandemic, nurses should engage in epidemiological screening and frailty assessment to identify high-risk groups and minimize transmission.
2. In the current COVID-19 pandemic, nurses should use technological devices for telehealth to reduce their direct contact with residents.
3. In the current COVID-19 pandemic, nurses should engage in communication and care planning with patients and relatives with regard to the current situation.

(c1-3) certainly fall within the scope of concrete recommendations for action. However, at first sight, it is not possible to rank these recommendations according, e.g., to their moral value. Any of the recommendations (c1-3) must be *prima facie* considered equally valid with a view to the underlying moral judgment (b3) (and hence with a view to the underlying moral principle (a)). This is what we call *contingency*_2_, designating the relationship between a moral judgment and the related recommendations for action as not being necessary but also not being impossible (and therefore not giving decisive reason to take a certain measure). Instances of contingency_2_ are of crucial importance in the realm of applied ethical considerations.

## Dealing with contingency_2_

Deriving recommendations for action from moral judgments is not possible by reference to purely formal considerations as in the case of inferring moral judgments from basic moral principles. Rather, the relation between moral judgments and recommendations for action is of a more material kind, i.e., certain ways of referring to empirical evidence are necessary to develop concrete recommendations. Contingency_2_ is primarily a result of the possibility to refer to different bridge principles in the process of inferring recommendations for action from moral judgments. A bridge principle is a non-trivial premise that—from the perspective of logical inference—together with at least one empirical premise and a moral judgment (Russ et al. 2006; Carnielli and Coniglio 2007; Mertz 2014) allows for concluding a specific obligation, permission or prohibition (e.g., a recommendation for action). Origins of the concept of bridge principles (or “bridge laws”, Nagel 1961) date back to the first half of the twentieth century and the debates around logical positivism. Aiming for a clear distinction of certain types of scientific sentences, logical positivists were faced with the question of how one type could be derived from the other. A way to explain this transition was to advocate for additional non-trivial premises encompassing features of both types of sentences (Carnap 1966). The idea was then prominently discussed by Hans Albert with regard to bridging the is-ought gap (Albert 1991). In the last decades, the concept has drawn increasing attention in the logic of ethical reasoning and bioethics (Schurz 1997; Russ 2002; Russ et al. 2006; Carnielli and Coniglio 2007; Paulo 2012; Mertz 2014; Romfeld 2019; Kuehlmeyer et al. 2022).

Since bridge principles merely describe a function within the reasoning process it is left to debate what exactly could serve as a bridge principle.^5^ For a recommendation like (c1), one could refer, e.g., to the Pareto principle, which (in the strong formulation) holds:

1. If harm should be prevented in a certain situation, and if carrying out X does not harm anybody, but not carrying out X harms some people, then X should be carried out.

Employed as bridge principle, (BP1) provides two empirically verifiable conditions for the claim that an action X that is for the good of some and does not harm anyone must be preferred over the alternative of not carrying out X. With regard to our case and existing empirical evidence it could be shown that, especially in confined spaces like nursing homes, asymptomatic infections are a driver of the pandemic (Ooi and Low 2020; Zhang et al. 2020). Strict epidemiological testing regimes including asymptomatic persons would allow to interrupt transmission at an earlier stage (Gostic et al. 2020). Earlier detection of asymptomatic carriers of the virus especially in high-risk groups would, thus, prevent at least some fatal outcomes. On the other hand, no evidence exists connecting tests for the coronavirus with any additional medical risk or harm for tested persons or for those who test, given suitable protective measures are put into place. With a view to moral judgment (b3) (and, hence, to moral principle (a)), the relevant empirical evidence as well as the Pareto principle, strict testing regimes could thus be recommended in a transparent way.

For (c2), it could be argued, e.g., by referring to a principle of comparable effectiveness. This principle states that (Raffée and Abel 1979):

(BP2) If X is better (more efficient) in achieving Z than Y (Y_1_, Y_2_, Y_3,_…,Y_n_), then X should be considered.

(BP2) states that those actions should be carried out by which a certain goal can be achieved better, i.e., more efficiently, than by alternative actions. If there is corresponding empirical evidence that the use of digital care technologies does not lead to a deterioration of care services and at the same time contributes to a reduction of contacts, this empirical evidence supports (c2) with a view to (b3) and (a). In this case, the use of digital care technologies would be a more efficient way since it is less associated with risks while resulting in identical outcomes. For example, it could be argued that tele-surveillance systems allow to observe and control routine care tasks without physical presence. Empirical evidence on some of these systems shows that the use of tele-surveillance systems is not associated with loss of quality of life in patients, can shorten hospital admissions significantly, and relieves care workers (Chopik 2016; Vincent et al. 2006).

As regards (c3), one could argue that concepts like healing presence, therapeutic use of self, intuitive sense, exploration of the spiritual perspective and patient-centeredness are at the core of nursing care. Conceptual analysis and empirical evidence show that almost all holistic concepts of nursing include the idea of an interactive process aiming at (re)establishing relationships, emphasizing positive aspects of the situation and nurturing self-care, thereby caring for the wellbeing of patients (Ramezani et al. 2014). Deriving (c3) would, thus, be to show that a certain task satisfies inherent goals of the functional role of nursing (Downing and Thigpen 1984) and must be pursued for this very reason. This is to employ what could be called a principle of telos stating that (Mertz 2014):

(BP3) Actions X (X_1_, X_2_, X_3,_…,X_n_) that further the realization of the inherent goal or purpose of a given practice P in a specific context are morally required.

According to (BP3) there is an obligation to act with a view to achieving a given *telos.* From statements about what certain practices actually *are* according to their telos as well as about ways to act accordingly, it is possible—in this view—to make evaluative statements about what someone ought to do as part of these very practices (Lawrence and Curlin 2011). In this case one might want to show that satisfying (c3) is an instance of caring for wellbeing which is connected to the inherent goal of nursing (and follows from (b3)) as well as (a)) and, hence, should be carried out.

It is important to understand that we do not advocate any of these arguments but rather use them for the purpose of demonstration. Furthermore, one may argue that there may be different recommendations for action which we failed to consider. However, the sole purpose here is to show how bridge principles and empirical evidence can be used to establish links between moral judgments (as instances of classes established by basic moral principles) and recommendations for action. We do not intend to develop substantive ethical arguments but aim at making evident that it is only possible to argue for or against certain recommendations for action, and, hence, to deal with contingency_2_ if their relation to empirical evidence has been explicated.

Finally, under ideal conditions, one would perhaps want to conclude that all the recommendations (c1-3) should be implemented. In real-world situations, however, this is often impossible. For example, necessary resources might not be available. Therefore, ethical argumentation usually does not end at establishing recommendations for action. Rather, further empirical evidence or evaluation of the reliability of the available information could provide indications that a certain recommended course of action is preferable over others, for instance, because it comes at fewer risks. This part of ethical argumentation is important, but not part of the framework presented here. We do not deny the importance of this step but rather understand the proposed framework as a prerequisite since critically assessing strengths and weaknesses of arguments as well as adding further considerations can only be carried out after the preconditions of respective recommendations for action have been argued clearly and transparently.

In addition, the proposed framework is rather ideally shaped. For example, empirical support for the crucial premises will always be partial and only be gradually plausible rather than proven. This inductive nature of empirical knowledge seems to create a certain tension with normative reasoning as proposed, which usually proceeds deductively from general to more specific assumptions and seems to be less open towards questions of epistemic uncertainty or of a possible underdetermination of evidence for its empirical premises. However, the framework does not preclude considerations about the strength and weakness of evidential support but explicates its role for normative reasoning. Implications, for example, of inductive argumentation is, in our opinion, a separate topic that, now, can be referenced as having importance for the credibility of the empirical premises in a normative argument, which could be another step to improve rigor of argumentation in empirically informed ethics.

## Implications for empirically informed ethics research

Our account of deriving ethically appropriate recommendations for action has several practical as well as theoretical implications for empirically informed ethics research. It could be claimed that contingencies may have serious implications for ethical recommendations as they may result in the impression of a certain arbitrariness of conclusions hampering their evaluation and adaption in practice. From a theoretical perspective based on our model of ethical reasoning, contingencies are an integral part of proceeding towards recommendations for actions that necessarily arise as the result of moral deliberation. However, we have also shown that overcoming these contingencies is far from being an act of arbitrariness but follows certain rules and necessities that can be derived from our model. While dealing with contingency_1_ might be a rather general problem known to different branches of normative ethics, we have highlighted that dealing with contingency_2_ is a specific task for applied normative ethics. For contextualizing moral judgments implies evaluating a certain normative requirement in light of a specific situation and, hence, requires, first, reference to relevant empirical knowledge as well as, second, introducing further substantial premises (i.e., certain bridge principles) to connect a moral judgment and the respective relevant empirical information.

This indicates the importance of bridge principles in empirically informed ethical reasoning, and at the same time raises difficult questions insofar as bridge principles in ethical reasoning have been largely neglected in theorizing about recommendations for action so far. We have defined bridge principles in accordance with existing literature as non-trivial premises that—from the perspective of logical inference—together with at least one empirical premise and a moral judgment allow for concluding specific obligations, permissions, or prohibitions. It must be noted, however, that there is no generally agreed definition of what constitutes bridge principles besides referring to its functional capacity of linking normative or evaluative with empirical premises in such a way that normative or evaluative conclusions become possible. This merely implies that bridge principles serve an important logical function. It does not, however, include any information or specification on how this function is served or what this implies for the quality and strength of a specific argument.

As our examples indicate, there seems to be a great variety of substantial additional premises that can be introduced as bridge principles (Kuehlmeyer et al. 2022) which, by their specific properties, substantially influence the recommendations one may be able to derive. Some of these principles may be uncontroversial, some may require more extensive scrutiny to show their soundness and acceptability (Kuehlmeyer et al. 2022). Some may, however, even carry additional normative weight and implications that might only be acceptable against the background of certain moral theories. A suitable example for this is the application of a principle of *telos* which allows to infer statements about what someone ought to do from what certain practices actually *are* according to their telos. This obviously implies understanding a telos in an Aristotelian fashion as something one ought to strive for. We, therefore, suggest that different bridge principles, depending on their properties may have different range, scope, and plausibility besides exercising their logical function as a connector. To use the metaphor of the bridge: it is by no means certain that all bridges are of the same size, quality, material, or durability. On the one hand, this shows that bridge principles decisively influence the strength and applicability of recommendations for actions while their theoretical structure and implications on the other hand are underexposed. The ambiguity and variety of bridge principles as well as their substantial contribution and importance to empirically informed ethical reasoning illustrates a gap at the intersection of is and ought that surely warrants more research regarding the structure and implications of different bridge principles and their use in ethical reasoning.

From a practical perspective, our considerations result in a shift of focus in terms of where the problem of contingencies and arbitrariness is situated. On the one hand, it seems to be quite usual that more than one recommendation for action can be derived in view of a specific morally relevant situation. On the other hand, however, it often seems to be the case that it is unclear *how* (by the use of what additional premises) these recommendations have been derived which, in turn, results in the impression of arbitrary recommendations for action. To our understanding, justifying and arguing for recommendations like (c1-3) involves not only explicating a basic moral principle like (a) and moral judgments like (b3), but also making explicit reference to that additional premise adopted to serve as a bridge principle. Given that transparency of scientific research and its reporting is a cornerstone of scientific debate, basic moral principles provide a point of reference for the further line of reasoning and help to establish a clear focus. Second, in specifying the formal relation between principles and moral judgments, it is crucial to be clear about the normative aspects in focus and possible alternatives. The general nature of basic moral norms and principles often allows for fundamentally different interpretations of values (like wellbeing or autonomy). The same applies to crucial conditions, addressees, and degrees of moral obligations. The use of bridge principles in justifying recommendations for action, however, poses the decisive link between empirical information and normative reasoning. It must be made explicit in research papers that combine empirical information and normative reasoning to allow recipients to understand the argumentation at hand (Kuehlmeyer et al. 2022). Finally, empirical evidence that is linked to the line of reasoning should be addressed in a precise way. The extent to which empirical data influences the reasoning via bridge principles should be critically discussed, and empirical hypotheses assumed to be true or testable in support of the argumentation should be stated. In this way, empirical research will be connected to other evidence and is opened for future empirical support or falsification.

## Conclusion

We started from the assumption that an ethical analysis committed to the goal of recommendations for action must undertake three conceptual steps. Nevertheless, the transition between these steps in current research is often implicit and creates the impression of arbitrariness in ethical reasoning. Against this background, we have developed a simplified model of ethical reasoning, showing that contingency is an inevitable consequence of ethical argumentation at this level, but does not necessarily result in arbitrariness. In this regard, it is important, however, as we have argued, to understand the role of bridge principles as non-trivial premises in ethical argumentation. The framework presented here, is, hence, a suggestion of a reconstruction of basic steps of argumentation in applied ethics. Second, it allows to develop ethically appropriate recommendations for action. Third, it allows to infer some recommendations for sound and transparent reporting of such argumentation and, hence, can also serve for critical argument analysis.

We do not deny that this model also has some shortcomings. It is certainly true that the underlying model of ethical reasoning is only a first and very rough approximation. Furthermore, we did not consider the role of specific legal situations, which may limit recommendations for action irrespective of whether these have been ethically justified according to our model. We also admit that there is more work needed to adequately conceptualize the concept of bridge principles in ethical reasoning, as this term surely encompasses a vast range of different non-trivial premises which warrant further careful inspection. However, it is also true that objections referring to these shortcomings only can be made due to the existence of a framework as we provided and would otherwise remain underexposed.

## Footnotes

1 This, of course, presupposes an is-ought gap.

2 For the purpose of our argument, we set aside the question here of how such basic moral principles or general norms can be justified appropriately.

3 Paulo (2012) for instance, understands moral judging as the process of developing a certain level of detail of a given moral principle in view of the circumstances in which it is to be applied. In other words, moral judging consists in the *specification* of moral principles in view of morally relevant decision situations; it is a rational process for narrowing the scope of a basic moral principle or norm by adding conditions of its application.

4 These are the recommendations we introduced in the beginning. For the sake of our argument, we will limit the following considerations to the contextualization of moral judgment (b3).

5 One of the major reasons to contest the usefulness of this concept is to point to this vagueness. Without following this debate in detail, we do not assume this to be problematic as long as a principle’s use is based in—and can be subject to—reason and is not a dogmatic assertion.
